# Fully Automated Segmentation of [^18^F]FDG and [^68^Ga]/[^18^F]PSMA PET/CT Images via Data-Centric Deep Learning

**DOI:** 10.2967/jnumed.126.272852

**Published:** 2026-11

**Authors:** Manuel Pires, Sebastian Gutschmayer, Enrica Bergalla, Stephane Chauvie, Dominik Amereller, Elisabetta Abenavoli, Aleksa Lazarevic, Natalia Wegrzyn, Philip Richter, Iustin Tibu, Ken Miller, David Kersting, Moon-Sung Kim, Jakob Dexl, Michael Ingrisch, Clemens C. Cyran, Maurice Heimer, Mej Kazazi, Jason Callahan, Rodney Hicks, Eduardo Calderón, Jonas Benecke, Fabian Schmidt, Roberto Sciagra, Julie Auriac, Romain-David Seban, Fanny Orlhac, Irene Buvat, Sophie Carina Siegmund, Liam Widjaja, Rudolf A. Werner, Giulia Santo, Ariane Kronthaler, Irene J. Virgolini, Yasser Gaber Abdelhafez, Hande Nalbant, Miju Cheon, Ramsey D. Badawi, Lorenzo Nardo, Anting Li, Ahti Vehmanen, Jukka Kemppainen, Riku Klén, Thomas Beyer, Lalith Kumar Shiyam Sundar

**Affiliations:** 1 QIMP Team, Medical University of Vienna, Vienna, Austria;; 2 Santa Croce e Carle Hospital, Medical Physics, Cuneo, Italy;; 3 Department of Biomedical Imaging and Image-Guided Therapy, Division of Nuclear Medicine, Medical University of Vienna, Vienna, Austria;; 4 Division of Nuclear Medicine, Azienda Ospedaliero Universitaria Careggi, Florence, Italy;; 5 Medical University of Vienna, Vienna, Austria;; 6 Department of Nuclear Medicine, West German Cancer Center, University Hospital Essen, University of Duisburg-Essen, Essen, Germany;; 7 German Cancer Consortium, Partner Site, University Hospital Essen, Essen, Germany;; 8 Institute for AI in Medicine, University Hospital Essen, Essen, Germany;; 9 Department of Radiology, LMU University Hospital, LMU Munich, Munich, Germany;; 10 Munich Center for Machine Learning, Munich, Germany;; 11 relAI–Konrad Zuse School of Excellence in Reliable AI, Munich, Germany;; 12 Melbourne Theranostic Innovation Centre, North Melbourne, Victoria, Australia;; 13 St Vincent’s Hospital, Department of Medicine, The University of Melbourne, Fitzroy, Victoria, Australia;; 14 Department of Nuclear Medicine and Clinical Molecular Imaging, University Hospital Tuebingen, Tuebingen, Germany;; 15 Werner Siemens Imaging Center, Department of Preclinical Imaging and Radiopharmacy, Eberhard Karls University Tuebingen, Tuebingen, Germany;; 16 IRIS, Institut Curie, Inserm, CNRS, Université Versailles Saint-Quentin, Universite PSL, Orsay, France;; 17 Department of Nuclear Medicine, LMU University Hospital, LMU Munich, Munich, Germany;; 18 Department of Nuclear Medicine, Medical University of Innsbruck, Innsbruck, Austria;; 19 Department of Radiology, University of California, Davis, Davis, California;; 20 Nuclear Medicine Unit, South Egypt Cancer Institute, Assiut University, Assiut, Egypt;; 21 Department of Nuclear Medicine, Veterans Health Service Medical Center, Seoul, Korea;; 22 Turku PET Centre, University of Turku and Turku University Hospital, Turku, Finland; and; 23 Department of Clinical Physiology and Nuclear Medicine, Turku University Hospital, Turku, Finland

**Keywords:** deep learning, PET/CT, tumor segmentation

## Abstract

The purpose of this study was to develop and validate lesion identification in oncologic nuclear imaging (LION), an open-source PET-only tumor segmentation pipeline for [^18^F]FDG and prostate-specific membrane antigen (PSMA)–targeted PET/CT, and to investigate how training data characteristics influence segmentation performance. **Methods:** In this retrospective multicenter study, 5209 [^18^F]FDG PET/CT scans spanning 19 disease types and 2046 PSMA-targeted PET/CT scans were used to train PET-only segmentation models. Tumor segmentation incorporated organs with physiologic uptake as auxiliary classes to enable PET-only inference. Tumor occurrence maps (TOMs) quantified tumor spatial diversity across the training data. For [^18^F]FDG, disease-specific and mixed-disease models trained on progressively larger subsets were compared to test whether increasing spatial diversity improves generalization. Scanner-related domain shift was analyzed using DINOv2 embeddings. Models were evaluated on multicenter holdout cohorts (616 [^18^F]FDG scans across 4 diseases; 443 PSMA-targeted prostate cancer scans) and compared with 3 open-source tools. **Results:** Organ context improved median Dice from 0.62 to 0.71 for [^18^F]FDG and from 0.75 to 0.83 for PSMA, on the complete holdout cohorts. Spatial diversity measured by TOMs was strongly associated with Dice (Spearman ρ = 0.80, *P* = 0.003). A mixed-disease model trained on 500 patients matched the performance of a lymphoma specialist model trained on 3031 cases. DINOv2 embeddings revealed scanner-induced domain shift between same-disease cohorts. LION achieved median Dice scores of 0.71 for [^18^F]FDG and 0.85 for PSMA and outperformed other open-source approaches on the model-comparison test set, which excluded the AutoPET test cases. **Conclusion:** LION enables PET-only automated segmentation for [^18^F]FDG and PSMA-targeted PET. Training data composition, particularly spatial diversity quantified by TOMs, was strongly associated with segmentation performance.

## Visual Abstract

**Figure:**
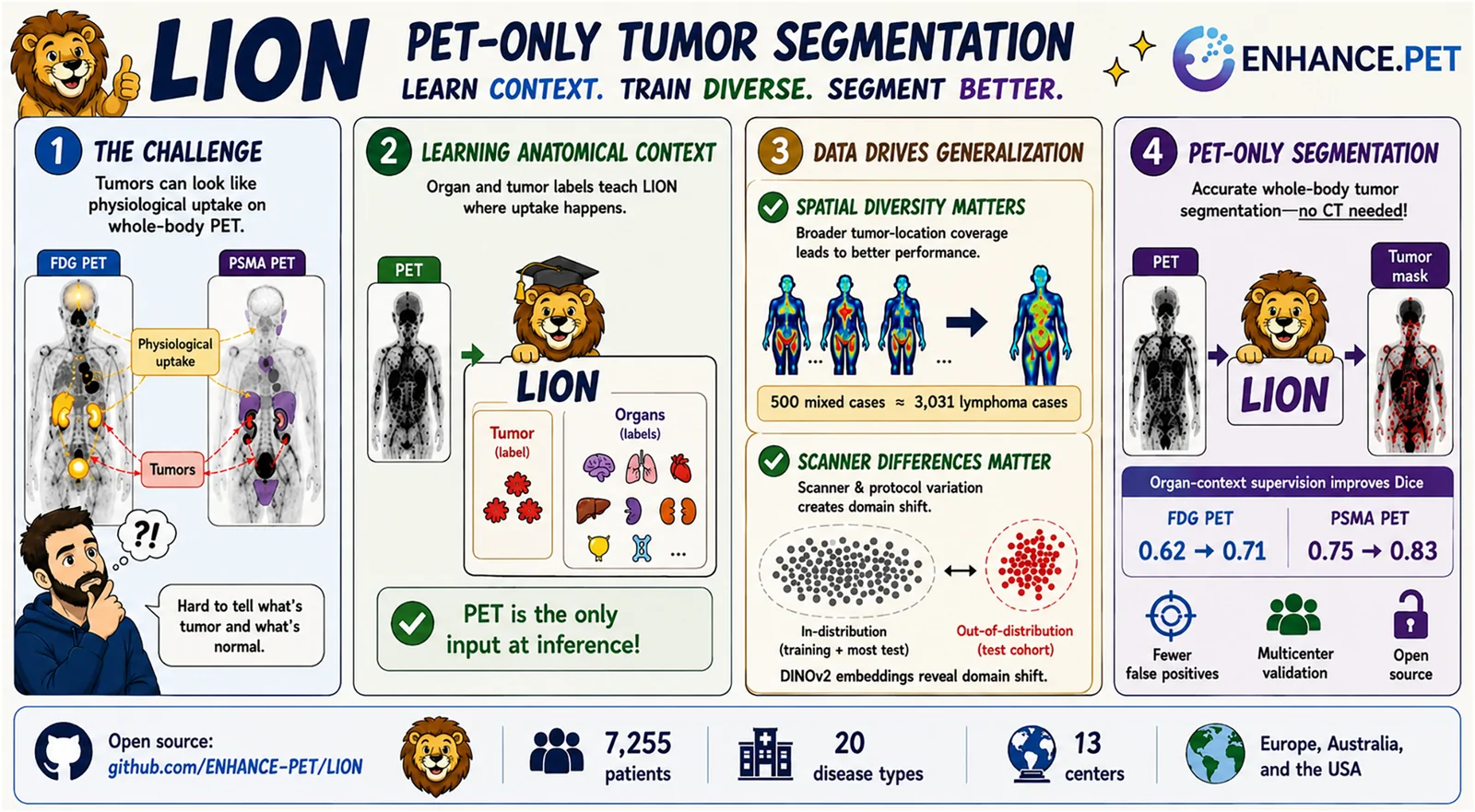

PET/CT is a cornerstone of oncologic imaging (1). [^18^F]FDG has served as the predominant radiotracer across cancer types, with FDG-derived total metabolic tumor volume (TMTV) increasingly recognized as a prognostic biomarker (2). In prostate cancer, radiotracers targeting prostate-specific membrane antigen (PSMA) have established PET as a theranostic modality by enabling staging and therapy selection for PSMA-targeted radioligand treatments (3,4). In this setting, quantitative assessment of whole-body molecular imaging tumor volume of PSMA-expressing disease and level of target expression is increasingly used to inform patient stratification and treatment monitoring. For both tracers, accurate quantification of tumor burden requires segmentation of PET lesions across the entire body. However, manual delineation can be time-consuming and impractical in routine clinical workflows, particularly in patients with widespread metastatic disease. As a result, segmentation remains a major bottleneck for translating PET imaging biomarkers into daily clinical practice (5).

Several automated PET segmentation approaches have been proposed, but most rely on paired PET and CT input at inference to provide anatomic context (6–8). Although effective, this design introduces a dependence on CT availability and quality which can reduce the robustness across heterogeneous clinical acquisition protocols. These limitations raise a fundamental question: is CT information truly required at inference, or can anatomic context instead be incorporated during training.

At the same time, recent advances in medical image analysis have emphasized increasingly complex architectures and large-scale foundation models (9–11). However, their utility is unclear when it comes to PET tumor segmentation. New PET tracers continue to emerge, often with significantly different uptake patterns compared with existing tracers, so that it remains uncertain how well the foundation models trained on current datasets will generalize to new tracer distributions.

Frameworks such as nnU-Net have demonstrated that carefully designed training pipelines can achieve strong performance across diverse segmentation tasks without extensive architectural modification (12). This motivates a data-centric deep-learning perspective, in which the quality, diversity, and structure of the training data are treated as primary determinants of performance rather than network architecture alone (13–15). Therefore, we hypothesize that current limitations in PET segmentation may stem less from model design and more from training dataset construction (16,17). Dataset composition is particularly important as variability in PET arises along 2 orthogonal axes. The first is disease distribution: across cancer types, the anatomic locations of tumors and their metastatic spread patterns can differ substantially. The second is scanner and protocol heterogeneity: differences in image quality, for example, between conventional and total-body PET systems, cannot be accounted for reliably through standard data augmentation or harmonization (18). Understanding how these factors influence model generalization is therefore likely to yield greater returns than further architectural complexity.

In this study, we introduce lesion identification in oncologic nuclear imaging (LION), an open-source software for enabling [^18^F]FDG and PSMA-targeted PET-only tumor segmentation. During training, CT-derived organ masks (16) are incorporated as auxiliary classes, allowing the network to learn anatomic context while remaining independent of CT at inference. We further examine 2 factors that may influence model generalization: disease distribution, quantified using tumor occurrence maps (TOMs), and scanner-/protocol-induced domain shift, assessed using DINOv2 (19–21) embeddings from PET maximum-intensity projections. These experiments were conducted on a large-scale, multicenter, multivendor annotated dataset of [^18^F]FDG and PSMA-targeted PET comprising 7255 patients and 20 disease types.

## MATERIALS AND METHODS

### Data

This retrospective multicenter study was approved by the institutional review boards of all participating institutions (Tables 1 and 2). Informed consent was obtained where required; otherwise, the requirement for consent was waived under retrospective approval.

**TABLE 1.** Training Data Overview: Summary of [^18^F]FDG and PSMA-Targeted PET/CT Training Datasets Used to Develop LION, Including Contributing Clinical Centers, Radiotracers, Number of Scans, Scanner Models, Disease Types, and Ethical Approval*

Center	Tracer^†^	No. of scans	Cancer type	Scanner models	Ethical approval
University Hospital Tuebingen	[^18^F]FDG	914	Lung cancer, lymphoma, melanoma, nononcological	Siemens Biograph mCT	Open source (27)
Medical University of Leipzig	[^18^F]FDG	370	Lung cancer	Siemens 1080; CPS 1080	Open source (28)
Azienda Ospedaliero Universitaria Careggi	[^18^F]FDG	193	Lung cancer	Philips GEMINI TF TOF 16; GE Discovery MI	Open source (28)
Institut Curie	[^18^F]FDG	108	Breast cancer	Siemens Biograph 20 mCT; Siemens Biograph 6 TruePoint; Siemens Biograph 64 mCT; GE Discovery 610; GE Discovery 690; GE Discovery 710; GE Discovery LS; Philips Guardian Body; Philips Ingenuity TF	DATA220207
Azienda Ospedaliera Hospital S. Croce e Carle	[^18^F]FDG	2631	Lymphoma	26 different Siemens models; 14 different GE models; 12 different Philips models; 2 different CPS models; Toshiba Aquiduo	NCT0355200; NCT0315989; NCT0206368; NCT0128774 NCT0079561; NCT0235431; NCT0433352; NCT0486665; NCT03964480
Universitätsklinikum Essen	[^18^F]FDG	993	Breast cancer, cancer of unknown primary, endocrine cancer, gastrointestinal cancer, gynecologic cancer, head and neck cancer, lung cancer, lymphoma, melanoma, neuroendocrine tumor, nononcologic, sarcoma, skin cancer (nonmelanoma), testicular cancer, thyroid cancer, urological cancer	Siemens Biograph Vision 600; Siemens Biograph mCT	24-12297-BO
LMU Klinikum	PSMA targeted	550	Prostate cancer	Siemens Biograph 64-4R TruePoint; Siemens Biograph mCT Flow 20; GE Discovery 690	Open source (29)
University Hospital of Tuebingen	PSMA targeted	193	Prostate cancer	Siemens Biograph mCT; Siemens Biograph Vision Quadra	627/2024BO2
Melbourne Theranostic Innovation Center	PSMA targeted	262	Prostate cancer	Siemens Biograph 128 Vision Quadra	2023-10-1292
Universitätsklinikum Essen	PSMA targeted	955	Prostate cancer	Siemens Biograph Vision 600; Siemens Biograph mCT	24-12297-BO
Medical University of Innsbruck	PSMA targeted	86	Prostate cancer	GE Discovery 690; GE Discovery MI	1296/2024

* [^18^F]FDG training data comprise 5209 patients spanning 19 cancer types; PSMA-targeted training data comprise 2046 prostate cancer patients imaged with a PSMA-targeted tracer across 5 centers.

**TABLE 2.** Holdout Data Overview: Summary of Independent Evaluation Cohorts Used for Model Testing, Including Center, Cohort Name in Article, Tracer, Number of Scans, Cancer Type, Whether Cohort Was Used for Open-Source Comparison, and Ethics Approval*

Center	Cohort	Tracer	No. of scans	Cancer type	Open-source comparison	Ethical approval
University Hospital Tübingen	AP-mixed	[^18^F]FDG	52	Lung cancer, lymphoma, melanoma	No	Open source (27)
LMU Klinikum	LMU-LC	[^18^F]FDG	369	Lung cancer	Yes	22-0416
(19)	Lym-JNM	[^18^F]FDG	58	Lymphoma	Yes	Open source (22)
University of California Davis	Lym-UCD	[^18^F]FDG	137	Lymphoma	Yes	1470016
LMU Klinikum	AutoPET	PSMA targeted	50	Prostate cancer	No	Open source (29)
Turku PET Center	Turku	PSMA targeted	50	Prostate cancer	Yes	T2025/10963
University of California Davis	UC Davis	PSMA targeted	173	Prostate cancer	Yes	2348464
Medical University of Innsbruck	Innsbruck	PSMA targeted	170	Prostate cancer	Yes	1296/2024

* [^18^F]FDG holdout data include 4 cohorts (AP-Mixed, LMU-LC, Lym-JNM, Lym-UCD; total *n* = 616) spanning multiple cancer types. PSMA holdout data include 4 prostate cancer cohorts (AutoPET, Turku, UC Davis, Innsbruck; total *n* = 443). Open-source cohorts denote publicly available datasets.

### Training Cohort

The [^18^F]FDG training cohort comprised 5209 PET/CT scans with clinically confirmed malignancy spanning 19 disease types, and the PSMA training cohort included 2046 prostate cancer studies imaged with a PSMA-targeted tracer. Detailed cohort characteristics, including participating clinical centers and scanner platforms, are summarized in Table 1. Tumor segmentation masks were generated by 13 board-certified nuclear medicine physicians following the annotation protocol described in the supplemental materials (available at http://jnm.snmjournals.org).

### Holdout Cohort

Eight independent holdout cohorts, completely disjoint from the training dataset at the patient level, were assembled for model evaluation (Table 2; Supplemental Table 1). For [^18^F]FDG imaging, 4 cohorts representing different cancer types and scanner platforms were included: AutoPET Mixed (AP-Mixed, *n* = 52, melanoma, non–small cell lung cancer, and lymphoma), LMU lung cancer (LMU-LC, *n* = 369, non–small cell lung cancer), JNM lymphoma (22) (Lym-JNM, *n* = 58, lymphoma), and UC Davis lymphoma (Lym-UCD, *n* = 137, lymphoma), for a total of 616 studies. To evaluate the effect of reference-standard definition across the [^18^F]FDG holdout cohorts, an exploratory harmonized analysis was performed by applying an SUV of at least 4 to both predicted and reference masks in AP-Mixed, LMU-LC, and Lym-UCD before recalculating Dice scores. Of note, Lym-JNM was not reprocessed because its evaluation standard already incorporated an SUV of at least 4. For PSMA imaging, 4 cohorts were included: UC Davis (*n* = 173), Turku (*n* = 50), AutoPET (*n* = 50), and Innsbruck (*n* = 170), totaling 443 scans.

### Model Architecture and Training

Two tracer-specific models were trained using the nnU-Net version 2 framework (12), one for [^18^F]FDG and one for PSMA-targeted tracers, with PET-only input. All PET scans and corresponding segmentation masks were resampled to an isotropic voxel size of 3 × 3 × 3 mm^3^ before training. Training hyperparameters, including patch size, batch size, network topology, and learning rate schedule, were automatically configured by nnU-Net on the basis of dataset characteristics. Models were trained for 1000 epochs using a combined Dice and cross-entropy loss. PET images served as the only input during training and inference. Training was performed using PyTorch version 2.5.1 (23) and nnU-Net version 2.5.1 on an NVIDIA A100 graphics processing unit with 80 GB memory. More information on the architecture and training can be found in the supplemental materials.

### Organ-Context Inclusion

CT images paired with each training PET scan were processed with MOOSE, an automated organ segmentation model (16), to generate organ masks that were combined with tumor masks to form multiclass training targets. For [^18^F]FDG, 10 auxiliary organ classes were included (brain, liver, spleen, kidneys, bladder, lungs, heart, pancreas, stomach, skeleton); for PSMA tracers, 5 classes were used (liver, spleen, kidneys, bladder, salivary glands). These organs correspond to physiologically high tracer uptake and represent major sources of false-positive tumor predictions (17). After inference, auxiliary organ outputs were discarded and only the tumor class was retained. The contribution of organ context was evaluated by comparing multiclass models with tumor-only models trained on the same cases and evaluated on the same holdout cohorts.

### Segmentation Performance Metrics

Segmentation performance was assessed using Dice similarity coefficient (DSC), lesion-level sensitivity, and positive predictive value (PPV). DSC quantified voxelwise overlap between predicted and reference tumor masks. Lesion-level sensitivity was defined as the proportion of reference lesions correctly detected by the model. PPV was defined as the proportion of predicted lesions corresponding to tumor lesions delineated by a physician.

### TOMs

To characterize the spatial distribution of tumors within the training data, we generated TOMs, 3-dimensional probability maps representing tumor occurrence in anatomic space across a cohort. For each case, the body mask and expert-annotated tumor mask were registered to a common anatomic template (24) and averaged voxelwise to produce cohort-level tumor probability distributions. Effective spatial coverage of a dataset was quantified using the inverse participation ratio:

$$C_{\mathrm{eff}}=\frac{1}{N}\times \frac{{\left(\Sigma_{i}p_{i}\right)}^{2}}{\Sigma_{i}{p_{i}}^{2}},$$ Eq. 1

where *p_i_* denotes the probability of tumor at voxel *i* and *N* is the total number of body voxels. This formulation quantifies the effective number of spatial locations represented in the dataset and discounts voxels with negligible tumor probability.

### DINOv2 Embedding Analysis

Using DINOv2 (19–21), a vision foundation model, embeddings were computed from coronal PET maximum-intensity projections and projected with UMAP (25) to identify holdout cohorts that deviated from the training distribution. This analysis was performed separately for [^18^F]FDG and PSMA-targeted tracers.

### Training Data Composition Analysis

To examine how training data composition influences segmentation performance, 11 [^18^F]FDG models were trained. Four specialist models were trained on single cancer types (breast, *n* = 229; melanoma, *n* = 212; lung, *n* = 833; lymphoma, *n* = 3031). Seven mixed models were trained on progressively larger subsets of the full cohort (*n* = 250, 500, 1000, 2000, 3000, 4000, and 5,209). Mixed-disease subsets were generated using nested sampling while maintaining center proportions; each successive subset added new cases to the prior subset such that the proportion of cases from each contributing site remained constant. All models were evaluated on the same 616 holdout patients (Table 2), enabling cross-evaluation across cohorts and paired comparisons between training strategies. Effective spatial coverage was computed for each model using TOMs. This experiment was restricted to [^18^F]FDG because the [^18^F]FDG training cohort spans 19 disease types with diverse spatial tumor distributions, whereas PSMA PET represents a single disease type.

### Open-Source Model Comparison

For [^18^F]FDG, LION was compared with 3 publicly available PET segmentation models: LesionTracer (6), Champalimaud (26), and TMTV-Net (7). All models were executed using their published default configurations on the same holdout patients. Models were compared using DSC, lesion-level sensitivity, and lesion-level PPV. LesionTracer and TMTV-Net used paired PET and CT input, whereas LION and Champalimaud operated on PET-only data. Model outputs were evaluated against the same reference masks (see Segmentation Performance Metrics). To avoid data leakage, AutoPET holdout cohorts (52 [^18^F]FDG and 50 PSMA-targeted scans; Table 2) were excluded because some competing models were trained on AutoPET challenge data, resulting in 564 common [^18^F]FDG and 393 common PSMA-targeted studies (Supplemental Table 1).

Because open-source models were evaluated on the same patients, head-to-head comparisons were performed using paired Wilcoxon signed-rank tests for Dice, lesion-level sensitivity, and PPV. The 95% CIs for median metrics and paired differences were estimated by bootstrap resampling over patients. *P* values were adjusted using the Holm method.

### Code Availability

LION is released as open-source software as part of ENHANCE.PET (https://github.com/ENHANCE-PET/LION). LION version 1.0.4 was used for this study. The exact trained weights evaluated in this article are available separately for the [^18^F]FDG model at https://github.com/ENHANCE-PET/LION/releases/download/lionz-v.1.0.3/clin_pt_fdg_5209_27032026.zip and for the PSMA model at https://github.com/ENHANCE-PET/LION/releases/download/lionz-v.1.0.0/clin_pt_psma_2046_25112025.zip. Any future model updates will be released separately and will not modify these archived weights. Training data are not publicly available because of institutional policies of contributing centers.

## RESULTS

We evaluated LION in 5 experiments: organ-context inclusion, holdout cohort evaluation, open-source model comparison, scanner domain shift analysis using DINOv2 embeddings, and training data composition using TOMs.

### Organ-Context Inclusion

Organ-context training substantially improved segmentation accuracy relative to tumor-only baselines for both tracers. Median Dice increased from 0.62 to 0.71 for [^18^F]FDG and from 0.75 to 0.83 for PSMA (Fig. 1A). Improvements were driven primarily by gains in PPV (from 0.50 to 0.67 for [^18^F]FDG and from 0.79 to 0.91 for PSMA), with unchanged sensitivity for [^18^F]FDG scans (0.86) and a slight increase for PSMA-targeted scans (0.88 to 0.93) (Supplemental Table 2).

**FIGURE 1.**
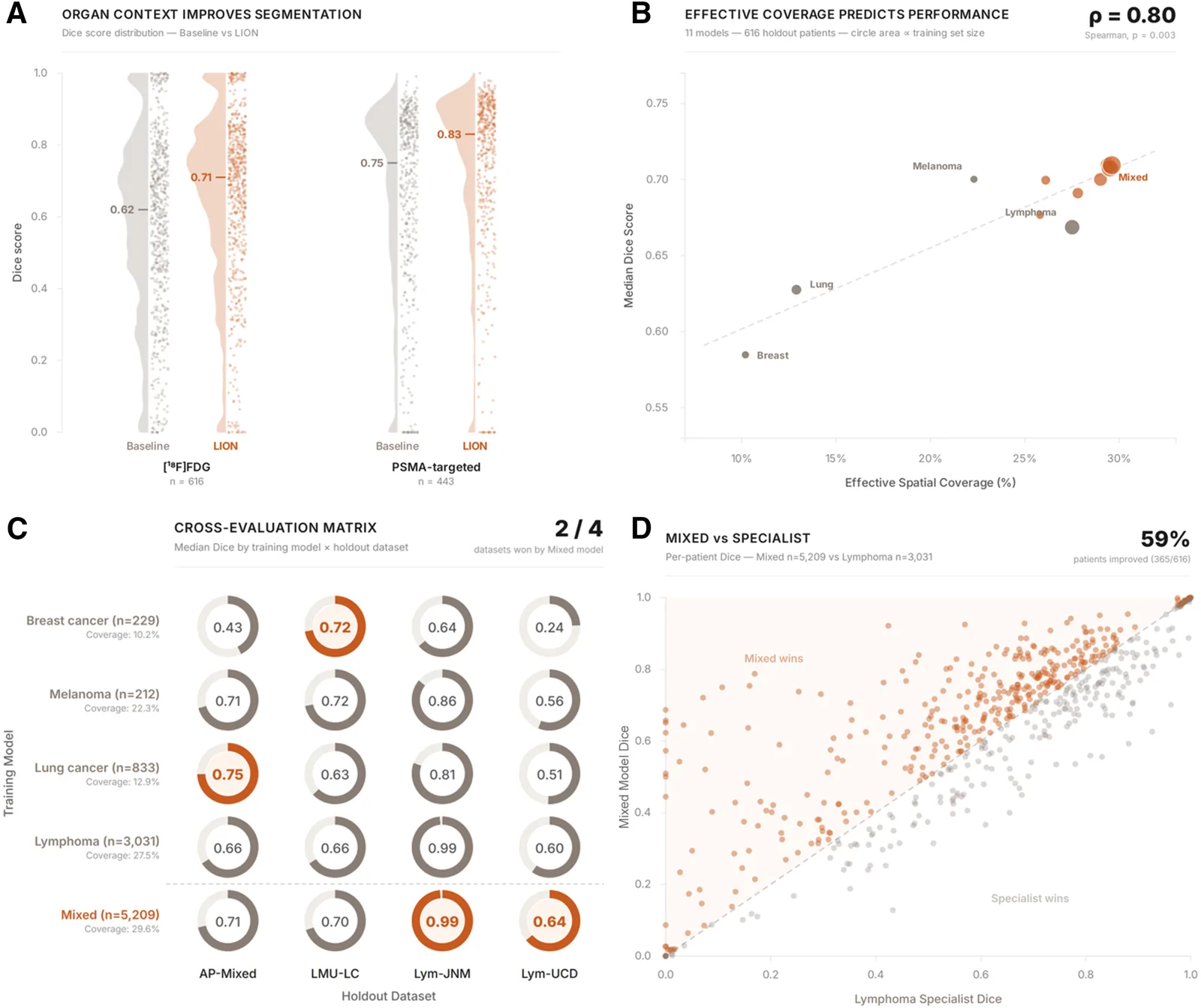
Organ-context ablation and training-data composition analyses. (A) Raincloud plots comparing Dice scores of baseline tumor-only (gray) and organ-context (orange) models on [^18^F]FDG (*n* = 616) and PSMA-targeted (*n* = 443) holdout cohorts. Half-violins show score distributions, jittered points correspond to individual patients, and horizontal bars denote medians. (B) Relationship between effective spatial coverage from TOMs and median holdout Dice across 11 [^18^F]FDG training models (4 specialist, 7 mixed). Marker size scales with training set size, and dashed line indicates fitted trend. (C) Comparison of mixed-disease model with disease-specific tumor-segmentation models trained exclusively on breast cancer, melanoma, lung cancer, or lymphoma cases, evaluated across four [^18^F]FDG holdout cohorts. (D) Per-patient comparison of full mixed model versus lymphoma specialist on [^18^F]FDG holdout set. Points above diagonal indicate improved performance with mixed-pathology training.

### Holdout Segmentation Performance

On 616 independent [^18^F]FDG holdout cases, LION achieved a median Dice of 0.71, a sensitivity of 0.86, and a PPV of 0.67 (Supplemental Table 3; Fig. 2A). Per-cohort median Dice scores were 0.71 for AP-Mixed, 0.70 for LMU-LC, 0.99 for Lym-JNM, and 0.64 for Lym-UCD (Supplemental Table 3). Bland–Altman analysis showed a volumetric bias of +36.58 mL (Supplemental Fig. 1A).

**FIGURE 2.**
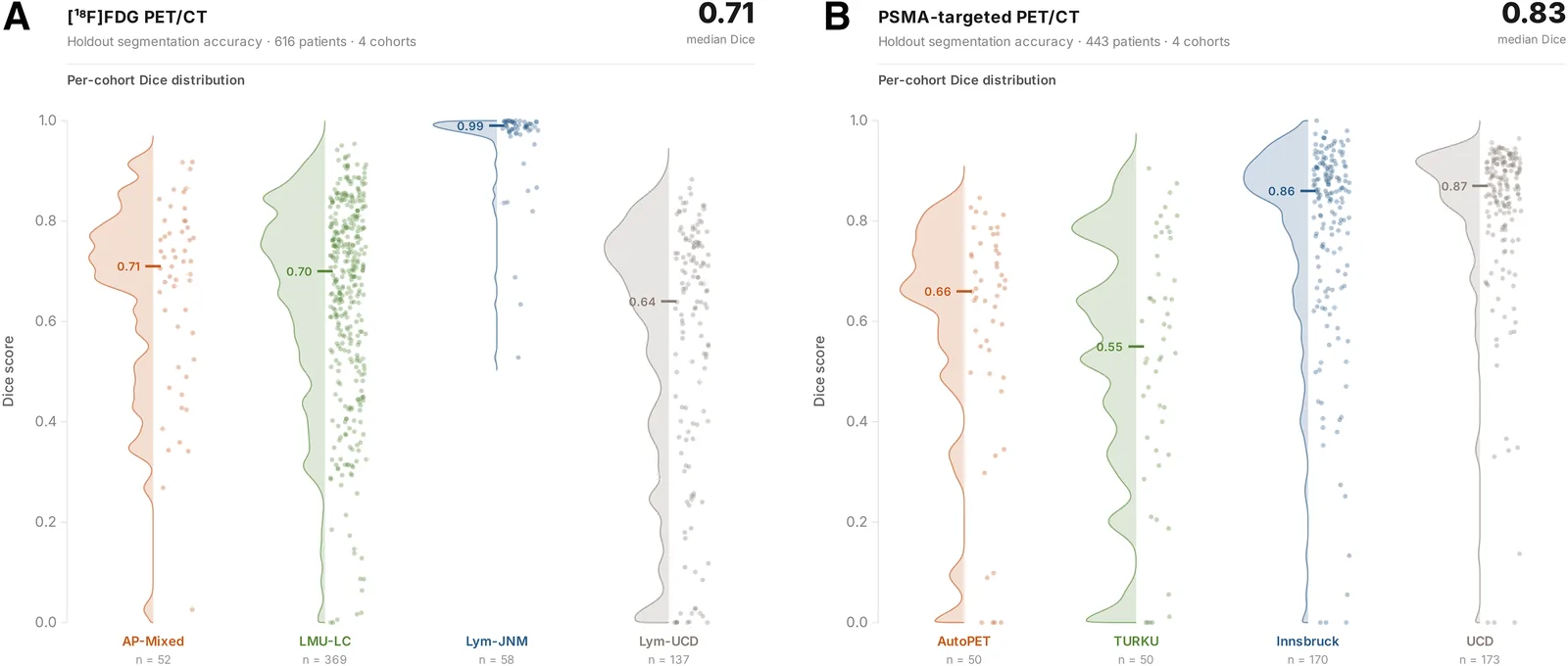
Holdout segmentation performance. (A) [^18^F]FDG holdout performance by cohort: raincloud plots showing Dice score distributions for AP-Mixed (*n* = 52), LMU-LC (*n* = 369), Lym-JNM (*n* = 58), and Lym-UCD (*n* = 137). Overall median Dice is 0.71. (B) PSMA-targeted holdout performance by cohort: UC Davis (*n* = 173), AutoPET (*n* = 50), Innsbruck (*n* = 170), and Turku (*n* = 50). Overall median Dice is 0.83.

Applying an SUV of at least 4 to both predicted and reference masks increased mean and median in AP-Mixed from 0.66 and 0.71 to 0.87 and 0.96, in LMU-LC from 0.65 and 0.70 to 0.83 and 0.91, and in Lym-UCD from 0.55 and 0.64 to 0.78 and 0.94, respectively. Lym-JNM, which already used an SUV of at least 4 evaluation standard, retained a mean Dice of 0.96 and median Dice of 0.99 (Supplemental Fig. 2). Across all 616 [^18^F]FDG cases, the SUV of at least 4 analysis yielded a mean Dice of 0.83 and a median Dice of 0.93.

On 443 independent PSMA holdout cases, LION achieved a median Dice of 0.83, a sensitivity of 0.93, and a PPV of 0.91 (Supplemental Table 3; Fig. 2B). Per-cohort Dice scores were 0.87 for UC Davis, 0.86 for Innsbruck, 0.66 for AutoPET, and 0.55 for Turku (Supplemental Table 3). In the Turku dataset, median patient-level lesion sensitivity remained at 1.00, whereas PPV was 0.67. Thirty-six of 50 patients had a metabolic tumor volume (MTV) below 10 mL; the median Dice was 0.48 in these patients compared with 0.75 in those with an MTV of 10 mL or greater (Supplemental Fig. 3). Bland–Altman analysis showed a mean volumetric bias of −3.29 mL (Supplemental Fig. 1B).

### DINOv2 Embedding Analysis

To investigate cohort-level performance differences in the holdout datasets, DINOv2 embeddings were computed from PET maximum-intensity projections and visualized using UMAP (Fig. 3). AP-Mixed, LMU-LC, and Lym-JNM overlapped with the training distribution, whereas Lym-UCD formed a distinct cluster (132/137 cases, 96.4%) (Fig. 3A). This separation partially corresponded to the reduced segmentation performance observed in Lym-UCD relative to the same-disease Lym-JNM cohort (median Dice, 0.64 vs. 0.99). In contrast, PSMA-targeted test cohorts showed no comparable separation from the training distribution (Fig. 3B).

**FIGURE 3.**
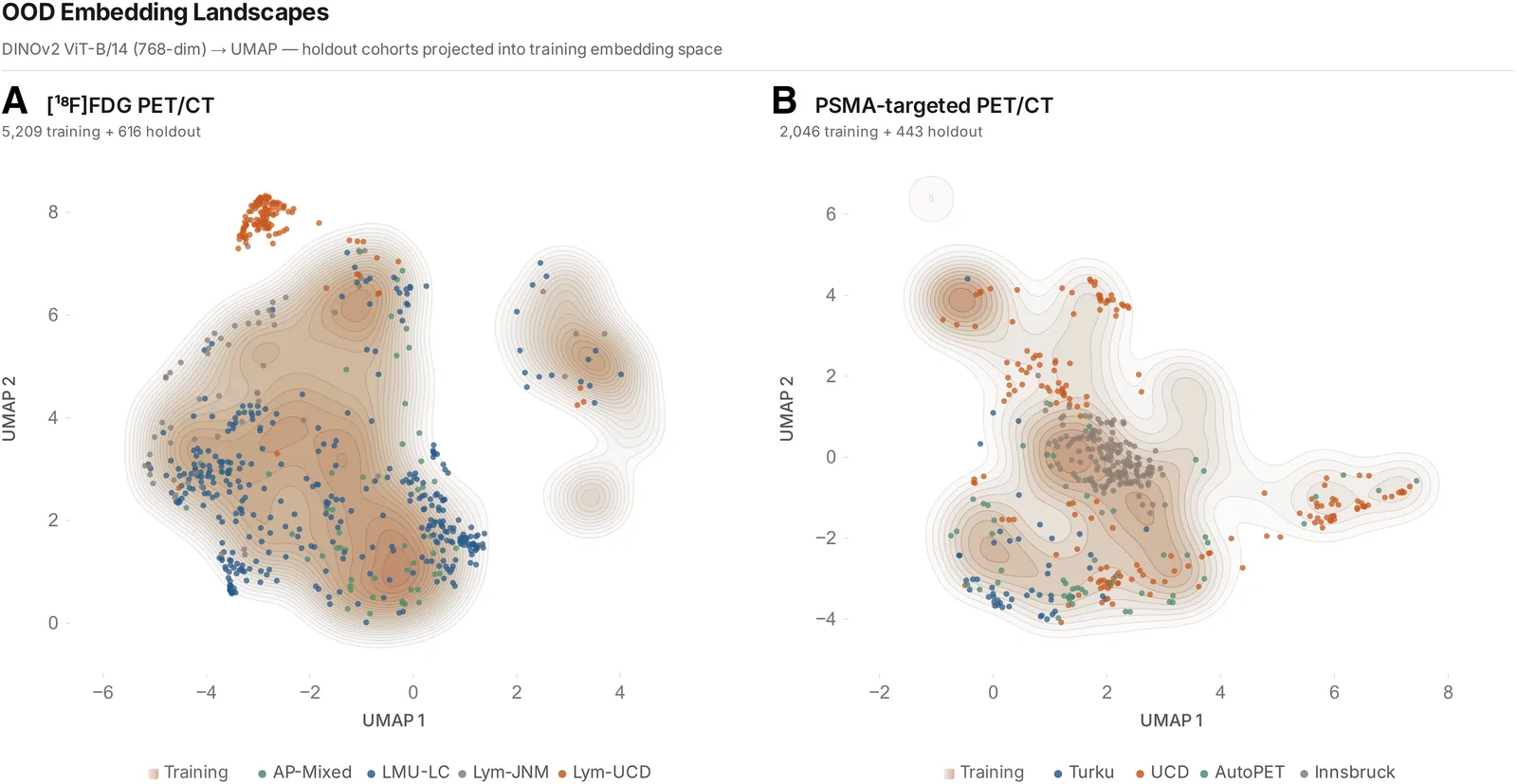
Joint FDG–PSMA embedding archipelago. DINOv2 ViT-B/14 UMAP projection of all [^18^F]FDG (A) and PSMA-targeted (B) training and holdout embeddings. Training data embeddings shown as density contours. Holdout cohorts colored by source. UC Davis FDG cohort (uEXPLORER total-body PET/CT; United Imaging Healthcare) forms distinct embedding island, with 96.4% of cases clustering separately from training data. OOD = out of distribution.

### Training Data Composition Analysis

TOMs derived from the [^18^F]FDG training data revealed distinct spatial tumor distributions across cancer types, with effective coverage of 28% of body voxels for lymphoma, 22% for melanoma, 13% for lung cancer, and 10% for breast cancer (Fig. 4; Table 3). Across the corresponding specialist models, median Dice on the [^18^F]FDG holdout set was 0.67 for lymphoma, 0.70 for melanoma, 0.63 for lung cancer, and 0.58 for breast cancer (Table 3).

**FIGURE 4.**
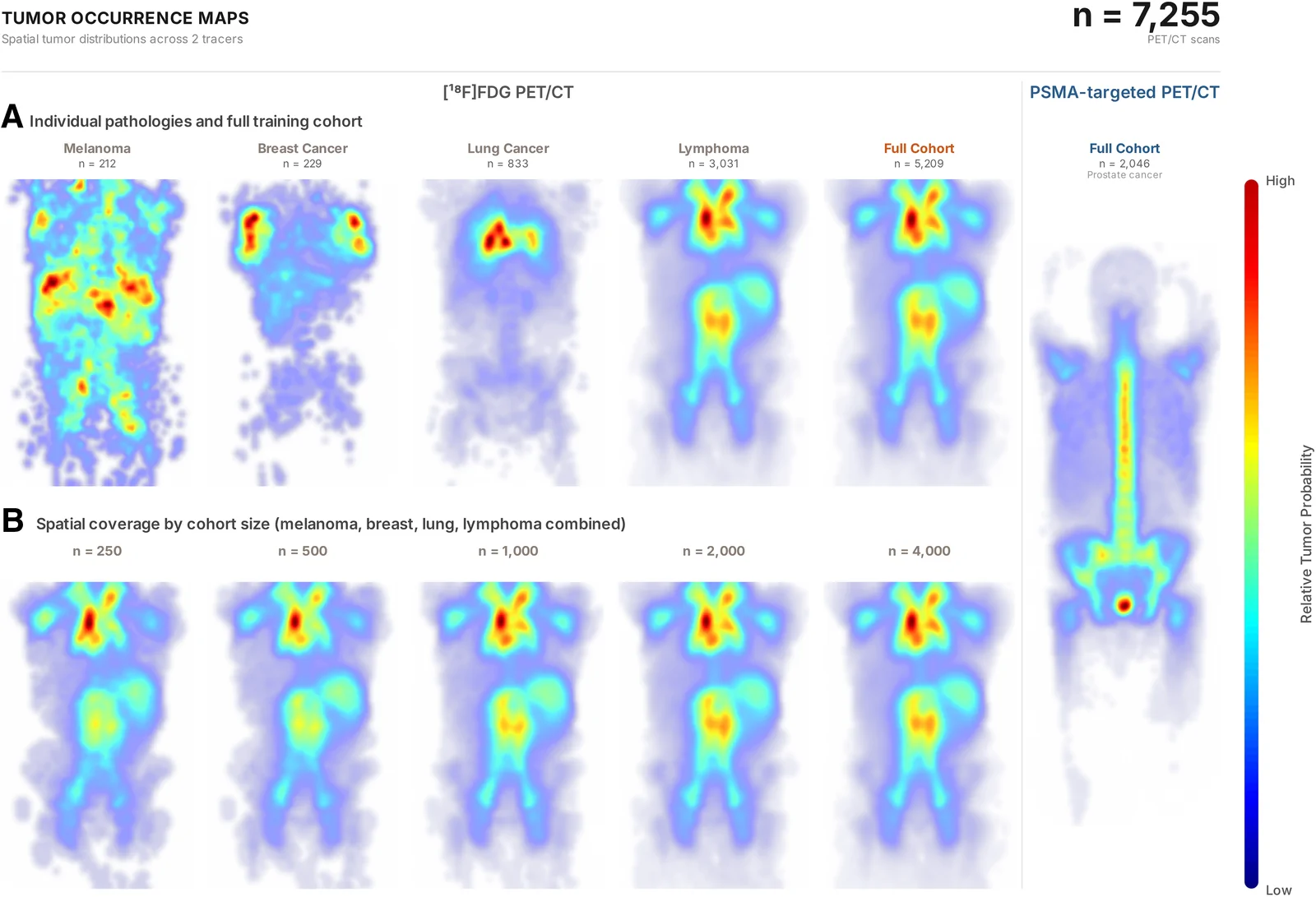
TOMs: spatial signatures of training data. (A) Individual pathology TOMs for 4 specialist models: melanoma (*n* = 212), breast cancer (*n* = 229), lung cancer (*n* = 833), and lymphoma (*n* = 3031). Coronal maximum-intensity projections with jet colormap show distinct spatial signatures. (B) Mixed cohort progression showing expansion of spatial coverage with increasing training size from *n* = 250 to *n* = 5209. (C) PSMA full cohort TOM (*n* = 2046) demonstrating characteristic pelvic and skeletal predilection of prostate cancer metastases.

**TABLE 3.** Cross-Evaluation: Median Dice by Model in each Dataset*

Training model	No. of cases for training	Coverage (%)	AP-Mixed	LMU-LC	Lym-JNM	Lym-UCD	Overall
Breast	229	10.2	0.43	**0.72**	0.64	0.24	0.58
Melanoma	212	22.3	0.71	**0.72**	0.86	0.56	0.70
Lung	833	12.9	**0.75**	0.63	0.81	0.51	0.63
Lymphoma	3,031	27.5	0.66	0.66	0.99	0.60	0.67
**Mixed**	**5209**	**29.6**	0.71	0.70	**0.99**	**0.64**	**0.71**

* Median DSC is reported for each model when evaluated on each [^18^F]FDG holdout cohort (AP-Mixed, LMU-LC, Lym-JNM, Lym-UCD), along with overall median across all [^18^F]FDG holdout scans. Training models include cancer-type specialists and full mixed-pathology model; for each model, number of training cases and effective spatial coverage derived from TOMs are shown to contextualize performance differences across training set compositions.

Mixed-disease models trained on progressively larger subsets showed modest improvements with increasing spatial coverage. From 250 to 4000 cases, Dice ranged from 0.68 to 0.71, whereas coverage increased from 26% to 29% (Fig. 4; Table 4). For 5209 cases, coverage reached 30% and Dice increased to 0.71 (Table 4). Across the 11 experimental models, effective spatial coverage was strongly correlated with holdout Dice (Spearman ρ = 0.80, *P* = 0.003) (Fig. 1B), whereas the log-transformed training set size yielded a similar correlation (ρ = 0.87, *P* < 0.001). Cross-evaluation of specialist and mixed models further highlighted the advantage of training on diverse disease distributions (Fig. 1C). The mixed model achieved the highest Dice on 2 of 4 holdout datasets.

**TABLE 4.** Mixed Model Scaling: Performance by Training Size*

No. of cases for training	Effective coverage (%)	Median Dice	Mean Dice
250	25.8	0.68	0.62
500	26.1	0.70	0.65
1000	27.8	0.69	0.64
2000	29.0	0.70	0.65
3000	29.4	0.70	0.66
4000	29.5	0.71	0.66
**5209**	**29.6**	**0.71**	**0.66**

* Performance of mixed-pathology [^18^F]FDG models trained on progressively larger nested subsets of full training cohort. For each training size, effective spatial coverage derived from TOMs is reported alongside median and mean DSC on fixed [^18^F]FDG holdout set (*n* = 616).

With lymphoma representing the largest single-disease training cohort (*n* = 3031), patient-level comparison was performed against the lymphoma specialist model (Fig. 1D). The mixed model improved Dice in 365 of 616 patients (59%), and when the analysis was restricted to lymphoma patients, the mixed model improved Dice in 121 of 195 cases (62%). Consistent with this pattern, a mixed model trained on only 500 cases achieved median Dice of 0.70, exceeding the lymphoma specialist model trained on 3031 cases (Dice, 0.67). In lymphoma cases only, median Dice was 0.70 for the mixed 500 model versus 0.69 for the lymphoma specialist. Similarly, the melanoma specialist model, despite using only 212 training cases but covering a broader spatial tumor distribution (coverage 22.3%), achieved a comparable Dice of 0.70.

### Open-Source Model Comparison

For comparison with existing models, analysis was restricted to the common evaluation subset described in the Materials and Methods (564 [^18^F]FDG scans and 393 PSMA-targeted scans; Supplemental Table 1). On the common [^18^F]FDG subset, LION achieved the highest median Dice, whereas LesionTracer achieved the highest PPV. Compared with LesionTracer, LION showed a small but statistically significant Dice improvement after Holm correction (0.71 vs. 0.68; median paired difference, 0.005; 95% CI, 0.000–0.018; Holm-adjusted *P* = 0.049), no significant difference in lesion-level sensitivity (0.89 vs. 0.89; Holm-adjusted *P* = 0.226), and lower PPV (0.67 vs. 0.78; Holm-adjusted *P* < 0.001). Thus, the [^18^F]FDG comparison reflects a trade-off between voxelwise overlap and false-positive burden rather than uniform superiority of one method across all metrics (Supplemental Tables 4 and 5; Fig. 5A).

**FIGURE 5.**
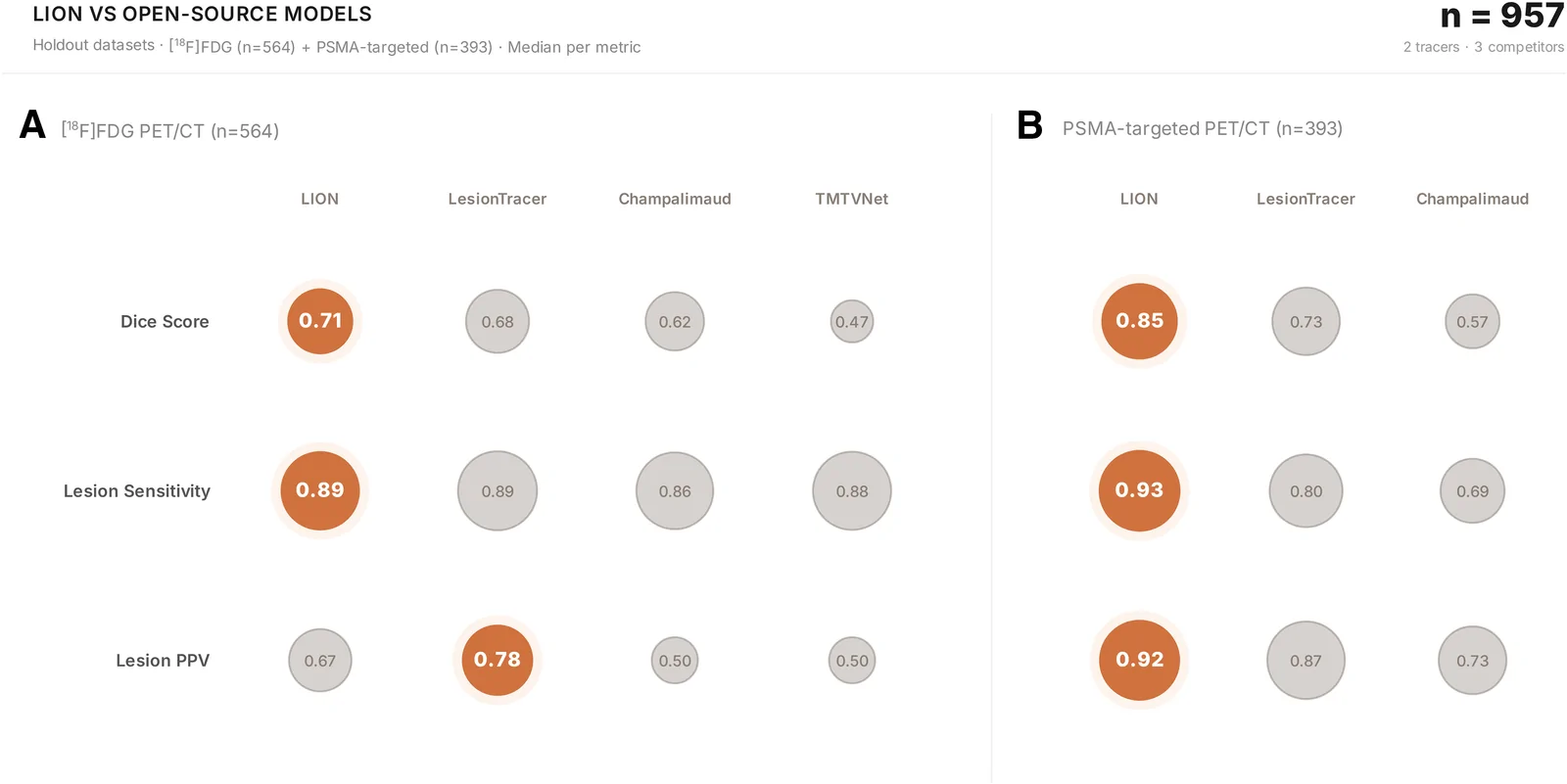
Open-source model comparison. (A) [^18^F]FDG comparison on 564 common holdout patients (AutoPET excluded to prevent data leakage): LION versus LesionTracer, Champalimaud, and TMTV-Net across Dice score, lesion sensitivity, and PPV. Bubble area proportional to metric value; orange highlights best performer per metric. (B) PSMA comparison on 393 common holdout patients: LION versus LesionTracer and Champalimaud. LION achieves the highest Dice (0.85 vs. 0.73) and lesion sensitivity (0.93 vs. 0.80).

On the common PSMA-targeted subset, LION achieved higher Dice than did LesionTracer (0.85 vs. 0.73; median paired difference, 0.096; 95% CI, 0.088–0.114; Holm-adjusted *P* < 0.001), higher lesion-level sensitivity (0.93 vs. 0.80; Holm-adjusted *P* < 0.001), and higher PPV (0.92 vs. 0.87; Holm-adjusted *P* < 0.001). Compared with the Champalimaud model, LION also achieved higher median Dice (0.85 vs. 0.57), lesion-level sensitivity (0.93 vs. 0.69), and PPV (0.92 vs. 0.73) (Supplemental Tables 4 and 5; Fig. 5B).

## DISCUSSION

We present LION, a PET-only tumor segmentation tool for [^18^F]FDG and PSMA-targeted PET, trained with organ context to learn anatomic guidance while remaining CT-independent at inference. This reduced false positives from physiologic uptake and increased median Dice from 0.62 to 0.71 for [^18^F]FDG and from 0.75 to 0.83 for PSMA. We further show that generalization is driven by training-data variability, quantified by TOMs (disease spatial diversity) and DINOv2 embeddings (scanner-induced domain shift). Across 6 independent multicenter holdout cohorts, LION achieved strong performance and the highest median DSC among evaluated open-source tools on common-patient subsets.

The organ-context ablation experiment clarifies the mechanism underlying the model’s performance. Adding CT-derived organ masks as auxiliary training targets during training increased [^18^F]FDG DSC from 0.62 to 0.71 and PSMA-targeted DSC from 0.75 to 0.83, with gains driven mostly by the reduction of false positives in areas of physiologic uptake, as shown by the improved PPV. This observation is reflected in the Champalimaud algorithm (26), which, for FDG, operates on PET-only input without organ-context supervision, achieving the same Dice as the tumor-only baseline model (0.62) (Figs. 1A and 5). Our ablation experiment indicates that PET-only inference with better segmentation accuracy is possible, provided the training is done with anatomic guidance.

With [^18^F]FDG, we intentionally favored mild oversegmentation to preserve flexibility for different downstream postprocessing strategies, for example, SUV of at least 4 postprocessing or an SUV of 2.5. PSMA performance varied across cohorts and was strongly influenced by tumor burden. The Turku cohort contained predominantly low-volume disease, with 72% of cases having an MTV of less than 10 mL compared with fewer than 20% at UC Davis. Despite its lower median Dice, Turku retained a median patient-level lesion sensitivity of 1.00, indicating that reduced overlap was not primarily due to systematic lesion omission. The lower PPV of 0.67 suggests that false-positive foci and imperfect contour agreement, amplified by very small lesion volumes, contributed substantially to the lower Dice. In addition, Dice is inherently more sensitive to small absolute volume differences, such that minor boundary deviations or additional small false-positive foci disproportionately reduce the score in patients with low tumor burden. Consistent with this, DSC increased with log-transformed tumor volume (ρ = 0.39, *P* < 0.001), suggesting that low absolute tumor burden was a key driver of PSMA segmentation performance in this setting.

The training data composition experiment highlights the importance of dataset diversity in tumor segmentation from PET imaging when it comes to generalization. Rather than annotating increasing numbers of cases within a single disease, performance gains appear when the training dataset covers a broader range of disease types. A mixed model trained on 500 cases achieved a median Dice of 0.70, matching the lymphoma specialist model trained on 3031 cases (Fig. 1B; Tables 3 and 4). This pattern persisted when restricting evaluation to lymphoma holdout cases only, where the mixed model performed comparably to the lymphoma specialist model. The melanoma specialist model showed the same pattern: 212 cases with a diffuse spatial distribution yielded a DSC of 0.70, matching the largest mixed subsets and outperforming the lung specialist model trained on 833 cases (Fig. 1B). For groups developing tumor segmentation models for emerging PET tracers, annotating a moderate number of cases across diverse cancer types may therefore be more effective than deep annotation within a single disease. The TOM framework allows this diversity to be quantified through spatial coverage of tumor occurrence across anatomic space.

The comparison between Lym-JNM and Lym-UCD highlights the combined influence of reference-standard definition and cohort-level acquisition differences. Applying a consistent SUV of at least 4 threshold to predictions and references substantially reduced the performance gap, indicating that annotation methodology explained much of the initial difference. The residual gap, together with the distinct Lym-UCD embedding distribution, is consistent with a broader scanner- and protocol-associated cohort shift.

Several limitations should be considered. First, a systematic double-read subset was not available across any contributing cohort; therefore, we could not establish study-specific interreader agreement or a performance ceiling for either tracer. Second, the [^18^F]FDG training data were dominated by lymphoma cases, which may bias the model. In addition, the [^18^F]FDG model has limited ability to distinguish inflammatory or treatment-related uptake from true tumors. Third, PSMA-targeted segmentation performance declines at very low tumor volumes, which correspond to clinically relevant decision thresholds in prostate cancer, particularly for patients that have biochemical recurrence. Fourth, the association between spatial coverage and segmentation performance observed in the TOM analysis is observational and does not establish causality. Moreover, the coverage metric is not yet prescriptive; it does not indicate how many additional cases or which diseases are needed to improve performance. Finally, LION currently provides no uncertainty estimates, limiting the ability to identify unreliable predictions at deployment.

## CONCLUSION

Organ-context supervision using CT-derived anatomic labels enables PET-only tumor segmentation to reduce false positives derived from physiologic uptake. DINOv2 embeddings identify scanner-related variability and characterize domain shift across acquisition platforms, whereas TOMs provide a framework for quantifying spatial diversity in PET segmentation datasets. The software is released as open source to support prospective validation.

## DISCLOSURE

Lalith Kumar Shiyam Sundar and Thomas Beyer are cofounders of Zenta GmbH/Austria. David Kersting reports a research grant from Pfizer; personal fees from Alnylam, GE HealthCare, Novartis, and Pfizer; and travel grants from Life Molecular Imaging and Sanofi, all outside of the submitted work. Rudolf Werner has received speaker honoraria from Novartis. Manuel Pires is funded by the Austrian Science Fund (FWF) under the Principal Investigators Project International program (Project I 6174). Sebastian Gutschmayer was supported in part by a research agreement between Siemens Healthineers and the Medical University of Vienna as well as by research funding from the Austrian Science Fund (FWF). Sophie Siegmund was supported by the Munich Clinician Scientist Program (MCSP). This work was supported by the French National Research Agency: ANR-22-CE45-0001 NEMO-PET and ANR-22-CE91-0011 INTER-ORGAN PET. Generative AI was used in the following tasks: Claude was used to assist with generating code for the plots in the article. Claude and ChatGPT were used to help generate codes for the tool; all codes were reviewed and tested by a human. ChatGPT was used to generate the graphical abstract. No other potential conflict of interest relevant to this article was reported.

## Notes

KEY POINTS **QUESTION:** Can PET-only deep learning enable accurate automated tumor segmentation in [^18^F]FDG and PSMA-targeted PET/CT, and how does training data affect performance? **PERTINENT FINDINGS:** In this retrospective multicenter cohort study, PET-only models trained on 5209 [^18^F]FDG and 2046 PSMA-targeted PET/CT scans achieved median Dice scores of 0.71 and 0.83, respectively, on independent holdout cohorts. Organ-context supervision improved Dice from 0.62 to 0.71 for [^18^F]FDG and from 0.75 to 0.83 for PSMA, and greater training-data spatial diversity was associated with better performance. **IMPLICATIONS FOR PATIENT CARE:** PET-only automated segmentation could support more practical whole-body tumor burden assessment.
